# The association between chronic liver diseases and preeclampsia

**DOI:** 10.1186/s12884-022-04827-4

**Published:** 2022-06-20

**Authors:** Sapir Nachshon, Eran Hadar, Ron Bardin, Shiri Barbash-Hazan, Adi Borovich, Marius Braun, Anat Shmueli

**Affiliations:** 1 Sackler Faculty of Medicine, Tel Aviv University, Tel Aviv, Israel; 2 Rabin Medical Center, Helen Schneider Hospital for Women, Beilinson Hospital, 39 Jabotinski St, 4941492 Petach Tikva, Israel; 3 Liver Institute, Rabin Medical Center – Beilinson Hospital, Petach Tikva, Israel

**Keywords:** Preeclampsia, Chronic Liver Disease, Hypertension

## Abstract

**Background:**

Preeclampsia is a multisystem disorder characterized by an abnormal vascular response to placentation associated with increased systemic vascular resistance. As liver involvement is one of the main clinical features of preeclampsia, we sought to determine if there is an association between chronic liver diseases and preeclampsia.

**Methods:**

A retrospective matched case–control analysis was conducted in a tertiary medical center. Three hundred eleven (311) pregnant women with preexisting chronic liver disease (study group), including viral and autoimmune hepatitis, non-alcoholic fatty liver, Wilson disease, and cirrhosis, were match for age, parity, and number of fetuses to 933 healthy pregnant women (control group). The primary outcome measure was the incidence of preeclampsia in each group. Secondary outcome measures were obstetrical and neonatal complications. Confounders found to be significant on univariate analysis were evaluated using logistic regression models, and odds ratios (OR) and confidence intervals (CI) were calculated.

**Results:**

Preeclampsia was diagnosed in 28 women (9.0%) in the study group and 33 women (3.54%) in the control group (*p* < 0.001).

On multivariate analysis adjusted for maternal age, parity, previous preeclampsia, chronic hypertension, gestational diabetes mellitus, pregestational diabetes mellitus, antiphospholipid syndrome, and mode of conception, chronic liver disease was found to be an independent risk factor for preeclampsia (aOR 2.631, 95% CI 1.518–4.561). Although there was no difference in the gestational week at delivery between the groups (38.6 ± 2.13 vs. 38.8 ± 2.17 for study and control group, respectively, *p* = 0.410), the study group had a lower mean neonatal birthweight (3088 ± 551 vs. 3182 ± 566 g, *p* = 0.011). There were no between-group differences in the other parameters evaluated.

**Conclusion:**

In our study, preexisting chronic liver disease was associated with a 2.6-fold increased risk of preeclampsia.

## Background

Preeclampsia is a multisystem disorder of unknown cause that is unique to human gestation. Although the pathophysiology is still not fully understood, it involves an abnormal vascular response to placentation associated with increased systemic vascular resistance, enhanced platelet aggregation, activation of the coagulation system, and endothelial-cell dysfunction [1]. Preeclampsia increases the risk of fetal, maternal, and neonatal mortality and morbidity including future cardiovascular disease.

The frequency of preeclampsia ranges between 2 and 7% in healthy nulliparous women [2, 3]. The frequency and severity of the disease are substantially higher in women with specific risk factors, such as multifetal gestation [4, 5], chronic hypertension [4, 6], previous preeclampsia [4, 7], pre-gestational diabetes mellitus [4], thrombophilia, lupus, and kidney disease [8]. Additional risk factors associated with preeclampsia are pre-pregnancy overweight/obesity, nulliparity, history of preeclampsia in a first-degree relative, prior pregnancy complications related to placental insufficiency (fetal growth restriction, abruption or stillbirth), maternal age ≥ 35–40 years, and conception by assisted reproductive technology [9].

Liver involvement, manifested as right upper quadrant abdominal pain or elevated transaminase levels, is one of the main clinical features of preeclampsia. This finding prompted several researchers to examine a possible association between preeclampsia and specific liver diseases, with conflicting findings. Some reported a higher incidence of preeclampsia in women with non-alcoholic fatty liver (NAFLD) and hepatitis B virus (HBV) infection [10–12] whereas others did not [13–16]. However, none of the studies to date investigated all chronic liver diseases at once, and all concluded that further research was needed. Therefore, the aim of the present study was to determine if chronic liver disease per se is associated with a higher risk of development of preeclampsia.

Showing a positive association between chronic liver diseases and preeclampsia would help to improve patient management and pregnancy planning and follow-up, thereby preventing maternal and neonatal morbidity.

## Methods

### Study population

A retrospective matched case–control study was conducted in a single tertiary medical center. The study group consisted of all pregnant women with a history of chronic liver disease who gave birth between July 2012 and August 2019. For the control group, we selected women without liver disease who gave birth during the same period and were matched 1-to-3 with the study group for age, parity, and number of fetuses. Only women with known pregnancy outcomes were eligible. Preexisting chronic liver disease in the study group was defined as hepatitis B carrier status, hepatitis C carrier status, primary sclerosing cholangitis, primary biliary cholangitis, autoimmune hepatitis, Wilson disease, or NAFLD.

Women less than age 18 years and women who were diagnosed with chronic liver disease after the index pregnancy were excluded from the study.

### Outcome measures

The primary outcome measure of the study was the incidence of preeclampsia during pregnancy and postpartum, with and without severe features. Secondary outcome measures were obstetrical and neonatal complications.

### Data collection

The data set was retrieved from the hospital’s comprehensive computerized healthcare database and included demographic and obstetric history, pre-gestational medical history, pregnancy complications, and pregnancy outcomes.

### Sample size

Sample size calculation was made using a statistical significance of 5% and a statistical power of 80%. The method that was used is a proportional comparison between two groups by a z-test. We assumed that the average rate of preeclampsia in the general population at our center is 5% and we hypothesize that the rate of preeclampsia among women with a chronic liver disease will be at least 2–3 times higher, according to previous publications. In order to show that the risk is doubled, the study group should contain 305 women and for a triple risk the study group should contain 100 women.

### Statistical analysis

Statistical analysis was generated using SAS, version 9.4. Continuous variables were presented by Mean ± Std, Categorical variables were presented by (N,%).

T-test was used to compare the value of normally distributed continuous variables between study groups, Wilcoxon was used for skewed continuous variables and Fisher's exact test was used to compare the value of categorical variables between study groups. Two sided p values less than 0.05 were considered statistically significant**.** Missing data were assumed to be missing at random, therefore only available data were analysed.

Confounders found to be significant on univariate analysis were evaluated using logistic regression models, and odds ratios (OR) and confidence intervals (CI) were calculated.

### Ethical approval

The study was approved by the local Institutional Review Board (approval number 0339–19-RMC).

### Definitions

The diagnosis of preeclampsia in a previously normotensive woman was based on the following criteria: new-onset hypertension (defined as systolic blood pressure ≥ 140 mmHg and/or diastolic blood pressure ≥ 90 mmHg, on at least two occasions at least 4 h apart; or systolic blood pressure of 169 mmHg or more or diastolic blood pressure of 110 mmHg or more, confirmed within a short interval of minutes) and proteinuria (defined as ≥ 0.3 g in a 24-h urine collection or protein/creatinine ratio ≥ 0.3 in a random urine specimen or random dipstick > 2 +) occurring after 20 weeks of gestation [17–19]. In the absence of proteinuria, preeclampsia was diagnosed if the new-onset hypertension was accompanied with the new onset of any of the following severe features: renal insufficiency (serum creatinine > 1.1 mg/dL or a doubling of the serum creatinine concentration in the absence of other renal disease), pulmonary edema, platelet count less than 100 X 10^9^/L, impaired liver function (elevated blood concentrations of liver transaminases to twice normal concentration, or new onset headache unresponsive to medication and not accounted for by alternative diagnoses or visual symptoms. [19]. Severe features of preeclampsia were defined as either systolic blood pressure ≥ 160 mmHg or more, or diastolic blood pressure ≥ 110 mmHg or more, on at least two occasions at least 4 h apart; platelet count less than 100 X 10^9^/L; renal insufficiency; pulmonary edema; new onset headache unresponsive to medication and not accounted for by alternative diagnoses, or visual disturbances; or impaired liver function that is not accounted for by alternative diagnoses and as indicated by abnormally elevated liver enzymes or by severe persistant right upper quadrant or epigastric pain unresponsive to medications.

The definition of severe features secondary to impaired liver function was changed in the updated ACOG Practice Bulletin No. 222 [19], replacing ACOG Practice Bulletin No. 202 [17], which was withdrawn.

Gestational age was calculated by the last menstrual period or by the first-trimester ultrasound if the discrepancy from the last menstrual period exceeded 7 days.

Fetal growth restriction (FGR) was defined as either estimated fetal weight below the 10^th^ percentile for gestational age or abdominal circumference below the 10^th^ percentile for gestational age, according to the local growth curves [20].

Gestational diabetes mellitus was diagnosed with the two-step approach. Gestational diabetes was defined as either a glucose challenge test of 200 mg/dL or higher between 24 and 28 gestational weeks or an abnormal 100-g oral glucose tolerance test with at least one pathological value, according to the criteria of Carpenter and Coustan [21].

## Results

The study group included 311 pregnant women with preexisting chronic liver disease and 933 matched women without liver disease. The distribution of types of liver disease in the study group is shown in Table 1; the most frequent was HBV infection (*n* = 143, 45.98%).

**Table 1:** Liver disease distribution

Liver disease	Frequency (N)	Percent (%)	PE **(N)**	PE Percent (%)
Hepatitis B	143	45.98	11	**7.69**
Unspecified hepatitis	64	20.58	4	**6.25**
Hepatitis C	51	16.4	4	**7.84**
Unspecified liver disorder	21	6.75	2	**9.52**
NAFL	12	3.86	4	**33.33**
Autoimmune hepatitis	7	2.25	0	**0**
Wilson's disease	5	1.61	0	**0**
Biliary cirrhosis	4	1.29	1	**25**
Cirrhosis	3	0.96	0	**0**
Unspecified viral hepatitis	1	0.32	0	**0**

*NAFL* Non Alcoholic Fatty Live

The demographic characteristics and medical-obstetrical history of the cohort are shown in Table 2. There were no significant between-group differences in maternal age, nulliparity rate, and gravidity. The study group had a higher rate of past fetal growth restriction (10.29% vs. 4.93%, *p* = 0.001) and previous miscarriages (0.53 ± 0.92 vs. 0.39 ± 0.82, *p* = 0.017).

**Table 2:** Baseline characteristics and obstetrical history of the study population

Parameter	chronic liver disease **(*n* = 311)**	without chronic liver disease **(*n* = 933)**	*p*-value
Age, years	31.82 ± 5.35	31.59 ± 5.22	**0.497**
BMI^a^	24.18 ± 5.23	23.97 ± 5.47	**0.703**
Gravidity	2.99 ± 1.73	2.83 ± 1.77	**0.188**
Parity	1.44 ± 1.33	1.39 ± 1.37	**0.589**
Fetus count			**0.847**
singleton	301 (96.78%)	906 (97.11%)	
twin pregnancy	10 (3.22%)	27 (2.89%)	
Spontaneous pregnancy^a^	263 (84.57%)	762 (81.67%)	**1**
Non-spontaneous pregnancy			**0.059**
IVF IUI	13 (4.18%) 2 (0.64%)	22 (2.36%) 1 (0.11%)	
Past cesarean section	0.28 ± 0.60	0.21 ± 0.57	**0.065**
Past miscarriage	0.53 ± 0.92	0.39 ± 0.82	**0.017**
Past FGR	32 (10.29%)	46 (4.93%)	**0.001**
Past IUFD	7 (2.25%)	9 (0.96%)	**0.081**
Past Preeclampsia	10 (3.22%)	18 (1.93%)	**0.189**

Data presented as mean ± standard deviation for continuous variables and as n(%) for categorical variables

*BMI* Body Mass Index, *IVF* Invitro Fertilization, *IUI* Intrauterine Insemination, FGR Fetal Growth Restriction, *IUFD* Intrauterine Fetal Death

^a^Data was missing in some of the women in the study groups

Table 3 presents the comorbidities of the cohort that are known risk factors for the development of preeclampsia. There was no major difference between the study and control groups in the prevalence of chronic hypertension (9.32% vs. 7.72%, respectively, *p* = 0.401). However, the study group had a significantly higher rate of pregestational diabetes mellitus (18.97% vs. 13.4%, *p* = 0.021), and obesity (6.75% vs. 0.54%, *p* < 0.001).

**Table 3:** Pre gestational medical history

Parameter	chronic liver disease **(*n* = 311)**	without chronic liver disease **(*n* = 933)**	*p*-value
Chronic HTN	29 (9.32%)	72 (7.72%)	**0.401**
Pre-gestational diabetes mellitus (type 1 + 2)	59 (18.97%)	125 (13.4%)	**0.021**
Obesity	21 (6.75%)	5 (0.54%)	** < .001**
SLE	2 (0.32%)	2 (0.21%)	**0.262**
Thrombophilia	1 (0.32%)	1 (0.11%)	**0.438**
APLA	1 (0.32%)	0 (0%)	**0.25**

Data presented as n(%) for categorical variables

*HTN* Hypertension, *SLE* Systemic Lupus Erythematosus, *APLA* Antiphospholipid Syndrome

Pregnancy complications and outcome are shown in Table 4. The study group had a significantly higher incidence of preeclampsia than the control group (9% vs. 3.54%, *p* < 0.001). On multivariate analysis adjusted for maternal age, parity, previous preeclampsia, chronic hypertension, gestational diabetes mellitus, pregestational diabetes mellitus, antiphospholipid antibody syndrome, and mode of conception, chronic liver disease was found to be an independent risk factor for preeclampsia (aOR 2.631, 95% CI 1.518–4.561).

**Table 4:** Pregnancy complications and outcome

Parameter	chronic liver disease **(*n* = 311)**	without chronic liver disease **(*n* = 933)**	*p*-value
Preeclampsia toxemia	28 (9%)	33 (3.54%)	** < .001**
Severe preeclampsia toxemia	8 (2.57%)	11 (1.17%)	
GDM	56 (18.01%)	85 (9.11%)	** < .001**
FGR	13 (4.18%)	40 (4.29%)	**1**
Preterm delivery (< 37 weeks)	30 (9.65%)	75 (8.04%)	**0.41**
Mode of delivery^a^			**0.09**
Vaginal	200 (64.94%)	648 (69.45%)	
Vacuum	24 (7.79%)	86 (9.22%)	
c-section	84 (27.27%)	199 (21.33%)	
Pregnancy week delivery	38.6 ± 2.13	38.8 ± 2.17	**0.41**
Augmentation of labor	22 (7.07%)	63 (6.75%)	**0.897**
Gender^a^			**0.075**
Male	171 (56.07%)	468 (50.16%)	
Female	134 (43.93%)	465 (49.84%)	
Weight (mean, grams)	3088 ± 551	3182 ± 566	**0.011**
Position^a^			**0.436**
Vertex	290 (94.46%)	892 (95.61%)	
Other	17 (5.54%)	41 (4.39%)	
1st minute APGAR score^a^			**0.42**
< 7	10 (3.27%)	23 (2.47%)	
> 7	296 (96.73%)	910 (97.53%)	
5th minute APGAR score			**1**
< 7	4 (1.31%)	12 (1.29%)	
> 7	302 (98.69%)	921 (98.71%)	
Perinatal death	2 (0.84%)	8 (0.86%)	**1**
NICU	1 (0.32%)	1 (0.11%)	**0.438**
PH	7.27 (6.57–7.97)	7.34 (7.27–7.41)	**0.114**

Continuous variables are presented either as median (range) or as mean ± sd; categorial values are presented n (%)

*GDM* Gestational Diabetes Mellitus, *FGR* Fetal Growth Restriction, *NICU* Neonatal Intensive Care Unit, *PH* Potential of Hydrogen, *APGAR* Appearance, Pulse, Grimace, Activity, Respiration

^a^Data was missing in some of the women in the study groups

Gestational diabetes mellitus was more prevalent in the study than the control group (18.01% vs. 9.11%, *p* < 0.001). However, on further analysis, after adjustment for various risk factors, including maternal age and body mass index, parity, preeclampsia in a previous pregnancy, chronic hypertension, antiphospholipid antibody syndrome, and non-spontaneous pregnancies, gestational diabetes was found to have a nonsignificant effect on the risk of preeclampsia (aOR 1.313, CI 0.636–2.709).

Although there was no difference in the gestational week at delivery between the groups (38.6 ± 2.13 vs. 38.8 ± 2.17 for study and control group, respectively, *p* = 0.410), the study group had a lower mean neonatal birthweight (3088 ± 551 vs. 3182 ± 566 g, *p* = 0.011). There were no between-group differences in any of the other pregnancy outcome parameters, maternal or neonatal, examined.

## Discussion

The present study showed that chronic liver disease was an independent factor for the development of preeclampsia, supporting our primary hypothesis.

Several studies have addressed the association between chronic liver disease and preeclampsia. Using a prospective design, Mousa et al. [10] showed that preeclampsia developed in 25% of 200 women with NAFLD compared to 14% of women without NAFLD. Memari et al. [11] reported a markedly higher prevalence of NAFLD in pregnant women with preeclampsia (66.7%) than in healthy pregnant women (23.8%), but noted that other variables, not evaluated in their study, may have affected this association. Ahmed et al. [12] conducted a case–control study on the association of HBV infection with preeclampsia., comparing 200 women with preeclampsia and 200 normotensive women. A significantly higher number of patients in the preeclampsia group were found to be HBsAg-seropositive, and the HBsAg-seropositive women had a 2.86-fold higher risk of preeclampsia than HBsAg-negative women. Others, however, reported contrary results. Keramat et al. [13] conducted a meta-analysis of 14 studies of preeclampsia and 6 of eclampsia comparing inactive HBV carriers and healthy pregnant women and found no between-group difference in the risk of preeclampsia and eclampsia. Bajema et al. [14] reported similar results in a retrospective population-based study of 4391 pregnant women with HBV infection and 22,410 seronegative pregnant women. Stokkeland et al. [15] examined the outcomes of more than 5000 births in women with HBV and hepatitis C virus (HCV) infection and found no higher risk of preeclampsia. Indeed, the risk of preeclampsia was decreased in the patients with HCV disease. The same group [16] also found no significant risk of preeclampsia in a nationwide study comparing 171 women with autoimmune hepatitis with a population control.

In a recent review of HBV carriers and pregnancy complications, Lao [22] addressed several studies examining the association of HBV infection with preeclampsia, eclampsia, and gestational hypertension. One study suggested a high incidence of preeclampsia among HBV carriers, 3 studies demonstrated a decreased risk of preeclampsia among HBV carriers, and 5 studies found no difference. The author speculated that the immune clearance phase of HBV infection resembles the immune response in preeclampsia and could therefore increase the risk of rejection of the conceptus and predispose the patient to preeclampsia. Thus, the conflicting findings among the studies might be explained by the phase of chronic infection in the different populations examined.

In our research, we referred to all chronic liver diseases as a group and calculated the overall risk of preeclampsia. Approximately 46% of all liver diseases were HBV infection and 16% were HCV infection. In contrast to some of the above-mentioned studies, we found that the incidence of preeclampsia was significantly more common in women with a chronic liver disease than in women without a known liver disease and that chronic liver disease was an independent risk factor for preeclampsia. The explanation of these findings relative to the literature may lie in our inclusion also of women with liver disease other than HBV and HCV, such as non-viral hepatitis. These different subtypes may have a stronger influence on the risk of preeclampsia. We may assume that women who have a chronic liver disease are more prone to the effect of a systemic disease that involves the liver than women without a chronic liver disease.

Another interesting finding of our study was the similar incidence of fetal growth restriction in the study and control groups. Given the higher rate of preeclampsia in the women with chronic liver disease, we would expect a concomitant increased incidence of this outcome as well. Further studies are needed to clarify this issue.

Ours is the first study to examine the association of preeclampsia with liver diseases as a group, and the results were established by multivariate analysis adjusted for various risk factors. The validity of evaluating liver diseases as a group is reinforced by the similar, well accepted, clinical approach used to study chronic kidney diseases and some inherited thrombophilia disorders in pregnancy.

By using the database from a single tertiary university-affiliated medical center, we were able to investigate a large cohort of women treated homogeneously with sufficient follow-up.

### Limitations

Our study has several limitations. First, owing to the retrospective design, data were missing in some cases, mostly body mass index and some newborn delivery outcomes. Second, also because of the retrospective design, we identified liver disease using the documented ICD codes and therefore may have missed some women with potential, still uncoded, liver disease or who were diagnosed in another hospital prior giving birth at our medical center. Third, more than 60% of the study group had viral hepatitis, with a much lower incidence of diseases of non-viral etiology, such as autoimmune hepatitis, Wilson disease, and biliary cirrhosis. This subdivision weakens our conclusions regarding these specific diseases.

## Conclusion

The findings of this study suggest that chronic liver disease per se is a significant risk factor for preeclampsia. We recommend considering close follow-up for symptoms and signs of preeclampsia in pregnancy women with a history of liver disease. To our best understanding, this may assist in the diagnosis of preeclampsia in its early stages and prevent life-threatening complications for both mother and newborn.

## Data Availability

The datasets used and/or analyzed during the current study are available from the corresponding author on reasonable request.

## Abbreviations

HBV: Hepatitis B virus
HCV: Hepatitis C virus
NAFLD: Non-alcoholic fatty liver disease
